# Can professional football clubs deliver a weight management programme for women: a feasibility study

**DOI:** 10.1186/s12889-018-6255-2

**Published:** 2018-12-03

**Authors:** Christopher Bunn, Craig Donnachie, Sally Wyke, Kate Hunt, Graham Brennan, Jemma Lennox, Alice Maclean, Cindy M. Gray

**Affiliations:** 1 Institute of Health and Wellbeing, College of Social Science, University of Glasgow, Glasgow, UK; 2 Social and Public Health Sciences Unit, Medical Research Council/Chief Scientist Office, University of Glasgow, Glasgow, UK; 3 Institute for Social Marketing, University of Stirling, Stirling, UK

**Keywords:** Weight management, Football, Women, Gender, Physical activity, Obesity

## Abstract

**Background:**

Levels of obesity remain high in the UK. The Football Fans in Training (FFIT) randomised controlled trial (RCT) demonstrated that a 12-week, gender-sensitised weight management, physical activity and healthy eating group programme delivered through professional football clubs helped men aged 35–65 years with BMI at least 28 kg/m^2^ lose a clinically-significant amount of weight. We aimed to test the feasibility of a minimally-adapted FFIT programme for delivery to women by assessing recruitment and completion rates; determining if the programme content and delivery required further refinement; and evaluating the potential of FFIT for Women to deliver improvements in weight and other clinical, behavioural and psychological outcomes.

**Methods:**

A feasibility study of the FFIT for Women programme including before-and-after measurements of clinical (weight, waist, body mass index [BMI], blood pressure) behavioural (self-reported physical activity, food and alcohol intake) and psychological (self-esteem, positive and negative affect, physical and mental HRQoL) outcomes at five professional football clubs. Post-programme focus groups assessed acceptability of the programme format, content and style of delivery for women.

**Results:**

Recruitment across the five clubs resulted in 123 women aged 35–65 years with BMI at least 28 kg/m^2^ taking part in the study. The mean weight (95.3 kg) and BMI (36.6 kg/m2) of the cohort were both suggestive of high risk of future disease. Of 123 women who started the programme, 94 (76%) completed it; 72 (58.5%) returned for 12-week follow-up measurements. Participants compared FFIT for Women favourably to commercial weight loss programmes and emphasised the importance of the programme’s physical activity content. They also spoke positively about group dynamics, suggested that the approach to food was less restrictive than in other weight loss approaches, and broadly enjoyed the football setting. Mean weight loss was 2.87 kg (95% CI 2.09, 3.65, *p* ≤ 0.001). Mean waist reduction was 3.84 cm (2.92, 4.77, *p* ≤ 0.001).

**Conclusion:**

In this evaluation, FFIT for Women was feasible, acceptable and demonstrated potential as a weight loss programme. Our findings suggest the programme has the potential to produce outcomes that are on a par with existing commercial and state-funded offerings.

**Electronic supplementary material:**

The online version of this article (10.1186/s12889-018-6255-2) contains supplementary material, which is available to authorized users.

## Background

Obesity remains one of the greatest global public health challenges [1]. In Scotland, 68% of adults are overweight or obese, and 29% fall into the obese category [2]. Associations between obesity and heart disease, type 2 diabetes and some cancers are firmly established, and projections suggest that by 2030, the cost of obesity could be as much as £2 billion per year in the UK [3]. Although some have criticised community-based weight management interventions, suggesting environmental interventions should be higher priority [4], it has been clear for some time that tackling obesity requires a multi-faceted approach [5].

Commercial and weight management programmes disproportionately attract women [6]. In 2010, the Football Fans in Training (FFIT) weight loss and healthy living programme was developed specifically to appeal to men [7]. FFIT is delivered in 12, weekly sessions at club stadia by trained club community coaches to groups of overweight and obese men. The programme is designed to be gender-sensitised in relation to context (the traditionally male environment of football clubs, men-only groups), content (information on the science of weight loss presented simply, discussion of alcohol and its potential role in weight management, ‘branding’ with club insignia) and style of delivery (participative and peer-supporting which encourages vicarious learning through interaction and positive ‘banter’). Each 90-min session combines classroom-based activities [8], including learning and practice of the behaviour change techniques (including an incremental pedometer-based walking programme) shown to be effective in improving physical activity and diet [9–11], with physical activity sessions. The balance of ‘classroom’ and physical activity sessions changes over the 12 weeks; later weeks focus more on physical activity as men become fitter.

In 2011/12, a randomised controlled trial (RCT) found FFIT to be effective at 12 months, with a mean difference in weight loss of 4.94 kg (95% CI 3.95, 5.94) in favour of the intervention group. There were also significant improvements in other objectively-measured clinical (blood pressure and waist circumference), self-reported behavioural (physical activity, diet, alcohol) and psychological (self-esteem, positive and negative affect) outcomes, and the programme was cost effective [7, 12, 13]. Recently, follow-up research has shown FFIT continues to be effective and cost effective long-term. At 3.5 years, men in the intervention group sustained a mean weight loss from RCT baseline of 2.90 kg (1.78, 4.02), significant improvements in other clinical, behavioural and psychological outcomes, and participation in FFIT was associated with an estimated incremental cost-effectiveness of £10,700–£15,300 per QALY [14].

As FFIT was rolled out for men across Scotland after the RCT, many clubs reported a demand for the programme to be made available to women. Women’s football is growing across Europe, with UEFA reporting an increase of ~ 1,000,000 registered female footballers between 1985 and 2015 [15]. As well as playing, women are also watching the game in larger numbers. In Scotland, 23% of surveyed male fans attended with their spouses or children [16]. This growth in women’s interest in football, combined with the national equality agenda and interest expressed by women, led the Scottish Government (which funds all deliveries of FFIT in Scotland) to commission a small number of FFIT for Women pilot deliveries in the 2014/15 football season. This study was conceived in response to this commission and aimed to explore the feasibility of a version of FFIT with minimal adaptions for women (FFIT for Women) through assessment of: recruitment rates; reasons why women attended; completion rates; and reasons for non-completion. It also aimed to determine if the intervention content and delivery were acceptable to women or required further refinement, and its potential to deliver improvements in weight, and in other clinical, behavioural and psychological outcomes.

## Methods

This feasibility study was conducted in five Scottish Professional Football League (SPFL) clubs between April and November 2014, and consisted of before-and-after measurements (at baseline and 12 weeks) of objectively-measured weight, and other clinical and self-reported behavioural and psychological outcomes, and post-programme focus group discussions.

### Recruitment

As the study was nested within routine delivery procedures at participating clubs, recruitment was conducted by the five clubs, with support from the research team [GB], using clubs’ pre-existing strategies for recruiting men. These included advertising the programme through club websites and social media, match day programmes, posters in the stadium and local community venues, and email shots to the club’s membership and community partners. Using these methods, clubs were asked to recruit up to 30 women aged 35–65, with a BMI ≥28 kg/m^2^, to ensure that each club had sufficient participants to fulfil the five deliveries commissioned by the funders. Women who expressed interest in the programme were invited to a baseline measurement session at which they provided informed consent to and enrolled in the study. A £20 voucher was offered to those completing the 12-week follow-up measurements.

### Initial adaptations: The FFIT for women programme

The FFIT for Women intervention was essentially the FFIT programme [7] with minimal adaptations to the content and format of delivery. In terms of content, UK dietary recommendations relating to ideal calorie and alcohol intake were changed to be appropriate for women (at that time UK alcohol recommendations for men were higher than those for women, although they are now equivalent [17]), and masculine pronouns were replaced with feminine pronouns throughout the coach delivery manuals and participant notes. In terms of format, clubs were asked to ensure that a female coach was present at each of the 12, weekly sessions alongside a male coach trained to deliver FFIT.

### Reasons for attendance, completion rates and reasons for non-completion

To assess reasons for attendance at the programme, a baseline self-report survey (see Additional file 1) included items that asked women why they wanted to join FFIT for Women. Respondents were asked to tick all options that applied to them, and an item assessing how often they watched football matches. To assess completion rates, coaches were asked to keep attendance registers for each of the 12 weeks of the programme. To assess reasons for non-completion, coaches were asked to keep a note of women who stopped attending programme sessions. An audio-recorded telephone interview was conducted with all non-completing participants who could be reached, and reasons for leaving the study noted.

### Acceptability

To explore the acceptability of the programme, focus group discussions were held with women who completed the programme at all five clubs after the 12-week programme ended. Participants were eligible to take part in a focus group if they had attended at least six FFIT for Women sessions. Focus groups (conducted by an experienced social scientist, who was known to participants due to previous contacts in the research process [AMc]) were held on club premises, were audio recorded with participants’ permission and were transcribed verbatim. We limited the number of participants to 8 from each club to ensure that all who joined a focus group had the opportunity to speak and be heard. The mean number of participants was 6, and the mean duration of the sessions was 58.8 min.

The topic guide covered reasons for joining FFIT for Women, what was liked and not liked about the programme, how it compared to other weight management programmes, and whether and why participants deemed the programme to be appropriate for women, even though it had originally been designed for men. Two researchers [CB and JL] read 5 transcripts and agreed a codebook, which JL applied, with quality checking from CB. Data were coded thematically [18] with reference to the topic guide. Through discussion, CB and JL constructed the broad themes used to present the findings of the thematic coding.

### Outcome data collection

To assess the programme’s potential to deliver improvements in weight and other outcomes, data were collected in club stadia at baseline and 12-weeks by fieldworkers trained in standardised measurement and questionnaire administration procedures. Weight (kg) was assessed using electronic scales (Tanita HD 352, Middlesex, UK), with participants removing shoes and emptying pockets prior to measurement. Height (cm) was measured using a portable stadiometer (Seca Leicester, Chino, CA, USA) with participants removing shoes before measurement. Waist circumference was measured twice (three times, if the first two measurements differed by 5 mm or more) and the mean of all recorded measurements was calculated. Resting blood pressure was measured after a five-minute resting period with a digital blood pressure monitor (Omron HEM-705CP, Milton Keynes, UK) and repeated twice when the first measurement was found to be > 130/90. The measurement equipment was calibrated prior to use.

The International Physical Activity Questionnaire Short Form (IPAQ-SF) [19] was used to assess weekly changes in self-reported total physical activity (scored according to the IPAQ scoring protocol [20]). An adapted [12] form of the Dietary Instrument for Nutrition Education (DINE) was used to assess changes in fatty, sugary and fruit and vegetable food consumption [20]. Alcohol consumption was measured using a diary that asked women to report the number of units they had consumed in the previous week [21].The Positive and Negative Affect Scale(PANAS) was used to capture self-reported changes in mood [22], and self-esteem was assessed using the Rosenberg Self-Esteem Scale [23].

### Statistical analysis

Paired t*-*tests (or Wilcoxon Signed Rank tests where assumptions of normality of distribution were not met) were used to assess changes in outcomes from baseline. T-tests and Chi-squared analyses were also conducted to test for baseline differences between those that returned for follow-up assessment and those who did not. All analyses were carried out using SPSS v21.

## Results

### Recruitment, who was attracted and why?

FFIT for Women attracted 123 participants, and each of the five clubs recruited sufficient participants to run a programme (*N* = 17–27). Time between recruitment and first delivery varied between 4 and 26 weeks, with the upper value attributable to two clubs’ facilities being unavailable during the 2014 Commonwealth Games. Women who enrolled in the study had an average age of 45.8 years, were mostly employed (84%) and classified themselves as White Scottish or White British (100%) (see Table 1). Many were at high risk of future disease: their mean weight was 95.3 kg (SD ± 17.7) and mean BMI was 36.6 kg/m^2^ (±6.9), which is considerably above the 30 kg/m^2^ threshold for obesity [24]. Mean waist circumference was 105.1 cm (±12.4). Blood pressure was within normal limits: mean systolic blood pressure (BP) was 126.4 mmHg (±17.1) and mean diastolic BP was 83.6 mmHg (±10.8).

**Table 1:** Baseline characteristics of participants in the FFIT for Women feasibility study

Physical measures	Mean ± SD (*N*)
Age (years)	45.8 ± 7.4 (123)
Weight (kg)	95.3 ± 17.7 (123)
BMI (kg/m^2^)	36.6 ± 6.9 (123)
Waist (cm)	105.1 ± 12.4 (122)
BP Systolic (mmHg)	126.4 ± 17.1 (116)
BP Diastolic (mmHg)	83.6 ± 10.8 (116)
Employment status	% (*N*)
In paid employment or self-employed	84.6 (104)
Permanently unable to work	4.1 (5)
Retired from paid work	2.4 (3)
Looking after home or family	7.3 (9)
Other	1.6 (2)
Educational attainment	% (*N*)
No educational qualifications	3.3 (2)
Standard grades or equivalent	19.5 (24)
Highers or equivalent	10.6 (13)
Vocational qualification	10.6 (13)
HNC/HND	22 (27)
First degree	20.3 (25)
Post-graduate qualification	8.9 (11)
Other	3.3 (4)
Missing	1.6 (2)
Marital Status	% (*N*)
Single	16.3 (20)
Married	54.5 (67)
Separated	1.6 (2)
Living with someone	17.9 (22)
Divorced	8.9 (11)
Missing	0.8 (1)
Housing Status	% (*N*)
Own outright	17.9 (22)
Mortgage or loan	57.7 (71)
Rent	22.0 (27)
Live rent free	0.8 (1)
Other	0.8 (1)
Missing	0.8 (1)
Ethnicity	% (*N*)
White British	22.8 (28)
White Scottish	77.2 (95)

**Fig. 1:**
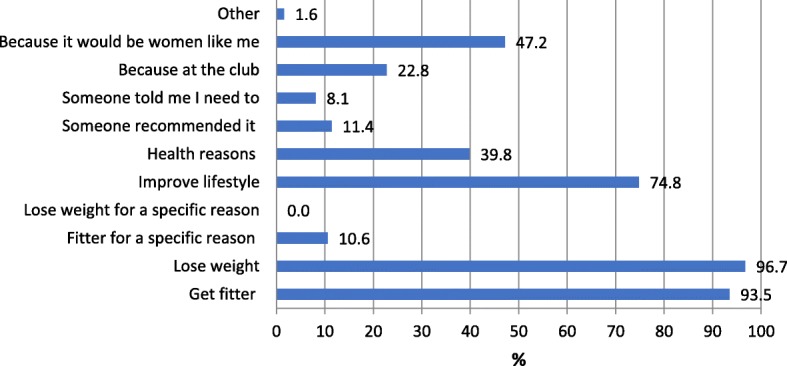
Reasons* participants joined FFIT for Women (*N* = 123) *NB women could cite multiple reasons

Figure 1 shows the main reasons that women reported for wanting to participate in FFIT for Women were to lose weight (97%), increase fitness (93%) and improve lifestyle (75%). The fact that the programme was aimed at ‘women like me’ (i.e. overweight/obese) also appeared to be important (47%), as did generic ‘health reasons’ (40%). Of less concern was the connection to the football club, which was seen to be important to only 23% of participants.

However, as Table 2 shows, the programme also attracted women who were actively engaged in local and televised football cultures: 35% reported attending all or most of their team’s home games, and 42% watched televised matches between 1 and 4 times a week. Within the cohort, 85% had not attended any form of weight management programme in the 3 months prior to joining FFIT for Women, but 47% had participated in a gym or leisure centre course, or attended the gym during this time.

**Table 2:** Football match attendance and viewing in FFIT for Women feasibility study

Attendance at home games	% (*N*)
I go to them all	27.5 (33)
I go to most of them	7.5 (9)
I go to some of them	39.2 (47)
I don’t go to any of them	25.8 (31)
Attendance at away games	% (*N*)
I go to them all	1.7 (2)
I go to most of them	10.8 (13)
I go to some of them	31.7 (38)
I don’t go to any of them	55.8 (67)
Watching games on TV	% (*N*)
Every day	2.5 (3)
5–6 times a week	5.0 (6)
3–4 times a week	16.7 (20)
1–2 times a week	25.0 (30)
Occasionally	45.0 (54)
Never	5.8 (7)
Watching games in a pub	% (*N*)
1–2 times a week	4.2 (5)
Occasionally	50.0 (60)
Never	45.8 (55)

### Attendance, completion rates and reasons for non-completion

**Fig. 2:**
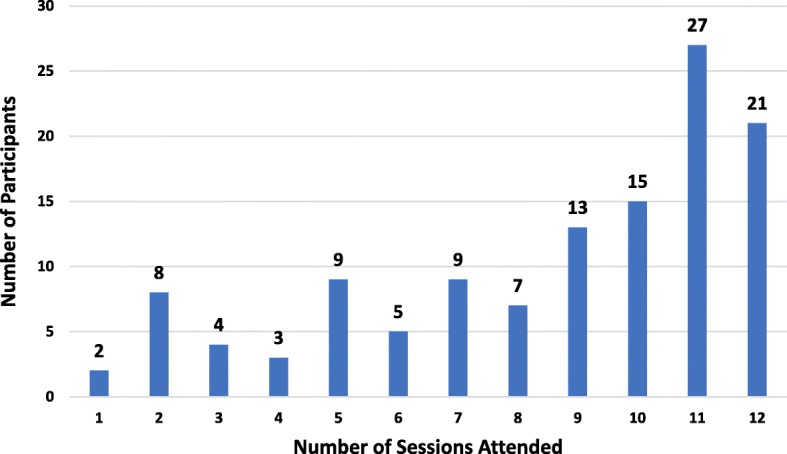
Number of FFIT for Women sessions attended by study participants (*N* = 123)

**Fig. 3:**
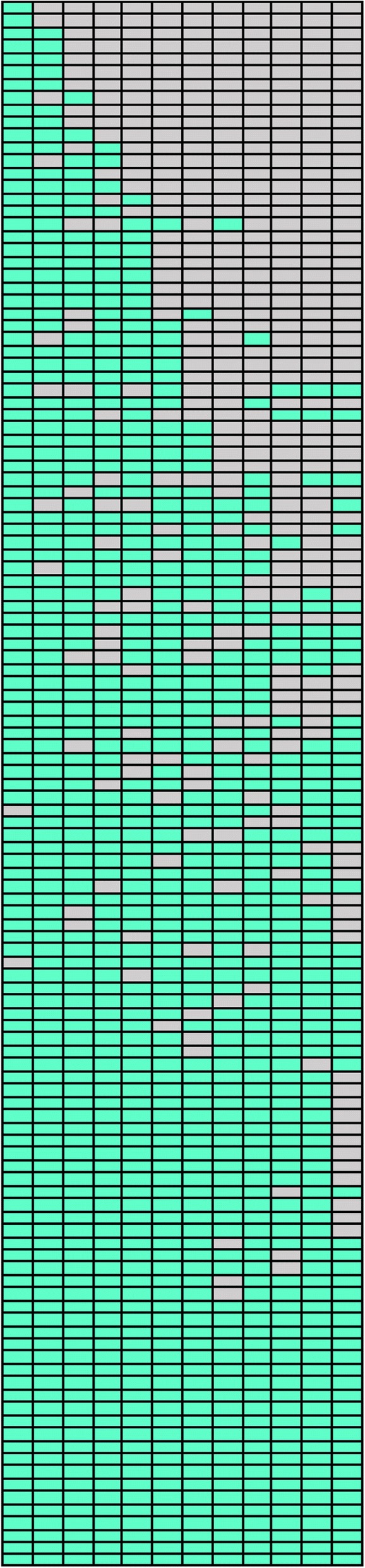
Visualisation of individual participant attendance, ranked in ascending order

FFIT for Women was well attended: of the 123 women who started the programme, 76% (94) completed it (attended 6 or more sessions) and 62% attended 9 or more sessions (see Figs. 2 and 3). Of the 29 women who did not complete the programme, only 7 were successfully contacted for a telephone interview. Two of these had stopped attending because of conflicting commitments, and one had experienced a bereavement. However, the non-completers were also critical of aspects of the delivery style, variously reporting that the physical activity sessions were too strenuous, and that the coaches lacked empathy and had been unprepared on occasion.

### Acceptability

The focus group discussions attracted 31 women across the 5 clubs. Findings suggest that the programme was mostly acceptable and are reported through four broad themes: comparisons to other weight management programmes (both in terms of physical activity and diet); reflections on the appropriateness of FFIT for delivery to women; and accounts of how women understood the group dynamics at play during the programme.

#### Comparison with other weight management programme - physical activity

In much of the focus group data, women emphasised how FFIT for Women was different to the weight loss programmes they had attended previously. The inclusion of physical activity in the programme was particularly important:

**P1:** This [FFIT for Women] puts a good emphasis on how much exercise plays… in part of your diet. I found that the diet clubs just put on… kinda the main emphasis on eating right. I mean eating right, yeah, that’s good, but you still need to do the exercise. ***C01_12wk.***

The physical activity aspects of FFIT for Women were viewed as enjoyable and sociable. One woman noted:

**P6:** I love the circuit – we all like the circuit-training. Wee bits some of us liked it, some of us didnae [didn’t]. And then we got boxercise the other night didn’t we [excited over-talk, confirming enjoyment]. ***C04_12wk.***

Another focus group exchange highlighted that it was the sociability of the exercise that made it enjoyable:

**P1:** See because you can talk [at FFIT for Women], when you go tae [to] these classes…

**P2:** They are funny though.

**P1:**… [At the other classes] it’s the person up there an’ there’s twenty people in the class an’ they’re shouting at you…

**P4:** Don’t know anybody.

**P1:**…telling you what to do an’ you’re just doing the exercise, exercise.

**P4:** I know, an’ they’re screaming at you.

**P1:** Here, we did the exercises but we were chatting to each other an’ enjoy, like having a laugh, didn’t we? We had a total laugh. ***C03_12wk.***

The inclusion of physical activity, then, was not only welcomed but it became valued part of the FFIT for Women programme, as well as space in which women could enjoy one another’s company.

Most, but not all, women also enjoyed the pedometer-based walking programme. For most women who took part in the focus groups, the pedometer was well received and seen to be a vital part of the programme:

**P8:** You know, but it makes you think. Oh my God. It gies [gives] you that bit mair [more] awareness o’ that side o’ it.

**P6:** Then you wantae walk more tae see how much you can do in a day, you know.

**P2:** That’s it, even a wee twenty minutes/half an hour of a lunch break I was doing that as well rather than sitting on Facebook, you know, at my lunch. ***C02_wk12.***

Using a pedometer allowed many women to become more aware of their levels of walking activity and encouraged them to build walking into their daily lives, including substituting it for sedentary lunchtime activities, such as using social media. However, a smaller number of women did not enjoy using the pedometer:

**P1:** I didn’t like the pedometer, I became obsessed with the step counting, I felt really down when I didn’t get the steps, so I took my pedometer off, because I knew I was still walking, but I just… it didn’t work for… didn’t work for me. ***C05_wk12.***

So while most women described the pedometer as motivating and useful, a small number did report that it was de-motivating and did not ‘work’ for them.

#### Comparison with other weight management programmes - diet

Another way in which focus group participants compared FFIT for Women to their past experiences of weight management, was through reflections on the structure of the dietary information that was offered.

**P3:** I think because it has been at our own pace an’ it’s not being dictated to.

**P2:** Mmhmm.

**P3:** That you can’t, you know, you’ve gottae have your two sins [local diet club term for ‘treats’] for this an’…

**P4:** You’re not rebelling.

**P6:** Probably enjoyed it better.

**P3:** … three points for that.

**P2:** Getting weighed every week.

**P6:** Aye.

**P3:** There’s not that worry about it that you can have the wee treats and not worry about them as much. ***C02_wk12.***

Whereas many commercial dieting clubs offer calorie-counting systems which came seem to ‘dictate’ food choices (e.g. through classifying some as ‘sins’), FFIT for Women offered more freedom with food, such that participants did not feel like they were ‘rebelling’ if they had a ‘treat’.

Other focus group participants emphasised that FFIT for Women offered them dietary advice that they could ‘trust’ and found useful as part of broad lifestyle change. For example:

**P1:** I find these slimming clubs… prey a bit on women. And I think they’re just money-making schemes so, yeah, I mean, I went to one, and I lost weight but… I found the advice I was getting from the leader of the class was just misleading, you know, that I was… I mean I’m not very tall, I’m not even, I’m 5 ft tall, but she was still telling me, “No, no, you must be… you can only be about 6 and half, 7 stone”, and I thought ‘no, that’s just too much’, and after advice from my GP, I thought ‘no, I’m not doing this anymore, I’ll find something that’s more…’, that’s proper basically, that’s not gonnae just try and say like, “If you buy these products that we sell, you’ll lose weight”, you know, I just… I don’t trust these slimming clubs at all.

**INT: Yeah, yeah. What about the rest of you then?**

**P2:** Well I’ve never, ever attended any slimming club, and don’t ever intend to either because… I like my food but… I feel they dictate to you what to eat, what not to eat, … and I don’t think… as you say, the money’s worth it either. So I was very pleased when I attended this [FFIT for Women], and it was the portions really that… that, you know, made me think… the size of the portions cause my husband, I think, he cooks, he’s retired and he does all the cooking, and… I was getting as big portions as him, which is about 4 times as much as I should be taking. ***C01_wk12.***

______.

**P6:** So it’s ongoing, it’s a work in progress and it’s a lifestyle change. It’s not a quick fix.

**P4:** It’s not a diet.

**P6:** It’s not a diet. It’s education to know how to improve your lifestyle, and to improve your life for you and your family. ***C04_wk12.***

In contrast to the experience of slimming clubs, FFIT for Women was valued because it was seen to be based on ‘proper’ evidence and free from commercial interest. In addition to this, focusing on portion sizes and lifestyle change was viewed as a useful part of the programme.

#### Appropriateness of the programme for women

As part of the focus groups, participants were asked to reflect on whether FFIT for Women needed to be adapted more to suit women. While some specific areas for improvement were raised, a common perception was that the programme was suitable as it was:

**P5:** The actual programme, itself, was – there was nothing in it different that us women couldn’t do. I don’t think it has to be different. I think the whole nutrition side of it, whether you’re a man or a woman, is the same, you know. It doesn’t, that doesn’t change. And the same with the fitness. ***C04_wk12.***

The perception of many participants was thus that the programme’s messages relating to nutrition and exercise are common to men and women.

One area in which participants felt the programme could be adapted to better suit women, related to menstruation.

**P8:** There was wan [one] kinda time when I think we were talking aboot [about] like obstacles and what gets in your way and things like that and what makes your like reach for the chocolate or something, and somebody was like that, “When you’re on your period.”

(laughter)

**P8:** “Oh I never had any o’ this on the man’s class.” [referring to male coach’s response].

(laughter)

**P8:** Oh he [referring to male coach] just buckled. [Female coach], she was hysterical, you know. And it’s things like that, an’ it’s like things like that, you need tae put in the book for women because it is.

**P4:** Aye.

**P6:** Yeah, aye, it’s...

**P8:** Everybody’s due on [due to start their period], an’ you’re like that, where’s the Cadbury’s or the Galaxy. Or whatever, you know, it’s...

**INT: Well that’s...**

**P8:** ... it is a thing that we dae [do]. ***C02_wk12.***

Focus group participants thus felt that the role played by menstrual cycles in women’s diet and physical activity habits should be acknowledged in the programme materials and discussions.

A second area in which change was suggested, so that the programme might better suit being delivered to women, related to body measurements.

P1: The measurement side of it, the only thing that I wish they’d done was tailored it to women. I wish they’d done your like proper waist measurement, and I wish they’d done your boob measurement, cause I would have liked to have seen how much I’d lost, cause I know my bras are smaller, and I would’ve liked to have known how much I’d lost off o’ [of] there, as opposed to like a kilt measurement, I’d like to know like my waist and boobs.

P6: Maybe your hips as well.

P1: Aye your hips, aye… your womanly bits. ***C05_wk12.***

The body size measurements taken by trained field staff at baseline and 12 weeks, and by coaches during the programme, focussed only on weight and waist. For some focus group participants, feedback relating to hips and bust would have been desirable.

#### Being part of a group

The group-based nature of FFIT for Women was widely reported to be an important feature of the programme. Pursuing weight loss with other women, doing the same things and facing similar challenges, was spoken about positively.

**P2:** Cause a lot of the walks at night, you got talking to somebody different from the week before.

**P3:** Yeah, you did.

**P2:** It was good. Each week it was somebody different.

**P3:** You know, it was good that way, it was good, you know, a good support from everybody, because everybody always said something that was encouraging or whatever, so it was good that way.

**INT: That’s good.**

**P1:** We got, I mean, we kinda used… kinda bounced our own ideas off each other as well, you know. “I’ve done this, what if you try it, see what happens”, so that’s good.

**P3:** It’s good when it’s in a group ***C01_wk12.***

The group-based nature of FFIT for Women offered participants the opportunity to get to know different people, provided support and offered a space in which they could discuss and share ideas.

Another aspect of the group setting that was mentioned during focus groups related to self-image:

**P3:** You know, and I’ve went tae [to] clubs, I’ve went tae classes there and I was, and I always felt, you know, I’m a middle-aged lady noo [now] and I always felt when I went intae [into] these classes it was full o’ young people and these exercises were so high impact an’, you know, an’ I felt like a burst couch within five minutes an’, you know, like. So I really felt like a fish oot ae [out of] water. ***C04_wk12.***

**____.**

**P4:** Yeah, yeah, yeah. ‘Cause I’m fed up o’ going into the gym and seeing someone that looks like a stick… stick insect, constantly in the gym all the time, it’s like ‘Why are you here? I’m here for a reason, why are you here?’, you know, and it’s quite intimidating as well, when you go down to the swimming, and you’ve got bumps and lumps sticking out … it was nice coming here, and everybody was the same. ***C05_wk12.***

Unlike some experiences of other group and/or gym-based exercise, FFIT for Women did not make participants feel overly self-conscious about their bodies (e.g. not feeling like a ‘burst couch’). The programme was also made up of people who were seen as being similar (e.g. with ‘bumps and lumps’), which added another positive dimension to the group dynamic.

A final dimension of the group experience in FFIT for Women was the common interest expressed by some participants in the football club at which the programme took place.

**P2:** I think all being likeminded, because we obviously all do, like, you know, obviously like [club name], you know, and that’s it, I think that’s got a big thing and coming in, you know, an’ you’re sortae inspired...

**P3:** Comfortable in your surroundings.

**P2:** Comfortable an’ you can speak to one another.

**P1**: But a lotta [lot of] the weeks, like especially us, we were all talking about the football when we come in on the Thursday we would all discuss last week’s game.

**P2:** What was that like, that was terrible, you know, whatever, you know.

**P1:** And, or whatever was in the newspapers that day (overtalk) you know, like so that broke the ice, the football thing definitely broke the ice with a lot of things ‘cause we all discussed...

**P3:** I think having the common bond I think that’s, I think that’s a big... ***C04_wk12.***

The football club, then, provided a talking point through which many women were able to bond. However, this was not something experienced by all participants. As one women put it “**P1:** I hate football. Can’t stand it. ***C03_wk12***.”

### Potential of FFIT for women to deliver improvements in outcomes

**Fig. 4:**
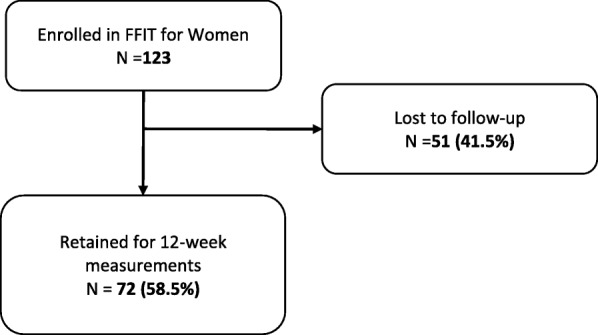
FFIT for Women Feasibility Study Flow Chart

Of the original 123 women enrolled in the evaluation study, 72 (58.5%) returned for 12-week follow-up measurements (see Fig. 4). We found no statistically significant differences between those who returned for follow-up measurements and those who were lost to follow-up (see Additional file 2). As Table 3 indicates, mean weight loss was 2.87 kg, *p* ≤ 0.0001 (95% CI 2.09, 3.65) and there were also statistically significant reductions in waist circumference, BMI and blood pressure. Table 4 also suggests the potential for the programme to deliver other benefits for women as evidenced by statistically significant improvements in self-reported physical activity, dietary and psychological outcomes.

**Table 3:** Change in objectively measured continuous outcomes (pre- and post-test)

	*N*	Mean Change (95% CI)	*p-value*
Weight (kg)	71	−2.87 (−2.09, −3.65)	≤0.001
BMI (kg/m^2^)	71	−1.11 (−0.79, −1.43)	≤0.001
Waist (cm)	70	−3.84 (−2.92, −4.77)	≤0.001
Systolic blood pressure (mmHg)	66	−8.08 (−4.11, −12.06)	≤0.001
Diastolic blood pressure (mmHg)	66	−5.15 (−7.98, −2.32)	0.001

**Table 4:** Changes in self-reported physical activity, psychological and dietary outcomes

	*N*	Baseline Mean (SD); Baseline Median (IQR)	Post-test Mean (SD); Post-test Median (IQR)	Mean Change (95% CI); Difference between medians (Z score)	*p-value*
Total Physical Activity (MET mins/week)	30	660 (442 to 1554)	1800 (966 to 2817)	1140 (−3.36)	0.001†
Fatty Food Score (Adapted DINE)	64	29.52 (9.01)	23.05 (7.81)	−6.47 (−8.56, −4.40)	≤0.001
Sugary Food Score (Adapted DINE)	64	5.11 (3.66)	2.30 (2.79)	−2.81 (−3.77, −1.86)	≤0.001
Fruit and Vegetable Score (Adapted DINE)	64	2.69 (1.49)	3.92 (1.61)	1.23 (0.81, 1.66)	≤0.001
Positive Affect (PANAS)	70	16.11 (3.63)	18.80 (3.62)	2.69 (1.76, 3.61)	≤0.001
Negative Affect (PANAS)	70	9.94 (3.68)	8.91 (2.75)	−1.03 (−1.83, −0.23)	0.012
Self-Esteem (Rosenberg)	71	17.66 (5.80)	20.3 (4.80)	2.63 (3.81, 1.46)	≤0.001
Alcohol consumption (units last week)	72	3 (0 to 10.75)	0 (0 to 8.88)	3 (−1.87)	0.062†

†Wilcoxon Signed Rank Test

## Discussion

Our feasibility study found that it was possible to deliver a minimally-adapted FFIT programme to women. The five participating clubs were able to recruit sufficient women to run the programme. Of the cohort, 76% (94) completed the programme. Focus group participants said that the physical activity component of FFIT for Women was appealing, new to many, and enjoyable, and compared the programme favourably to experiences in other weight management settings. The dietary component of FFIT for Women was also compared favourably to some commercial offerings, with participants suggesting they felt less ‘dictated to’. Focus group participants suggested that they enjoyed being part of a group and that the programme did not require much adjustment for future deliveries, but did raise menstrual cycles and additional body measurements as areas for consideration. Finally, the programme demonstrated potential to deliver positive outcomes at 12-weeks, with an overall mean weight loss of 2.87 kg.

Below, for context, we make some comparisons between changes at 12 weeks in this small-scale study of FFIT for Women, and the 12 week outcomes of men who participated in FFIT during the 2011/12 RCT [12]. These should be interpreted with caution, not least because of the difference in completeness of follow-up, but they present some interesting reflections to be explored in future work. When compared to men in the FFIT RCT (mean age 47.1 years, mean BMI 35.3 kg/m^2^) [12], FFIT for Women attracted participants with similar mean age (45.8 years) and BMI (36.6 kg/m^2^). These findings confirm that FFIT for Women shows potential to reach the intended population, i.e. women whose elevated BMI puts them at heightened risk of future ill-health. However, while the FFIT RCT demonstrated that participants achieved a mean weight loss of 5.80 kg [95% CI 6.33, 5.27] at 12 weeks (and 5.56 kg [4.70, 6.43] at 12 months), FFIT for Women, at least in these early deliveries, appears to deliver lower levels of weight loss (mean 12-week weight loss 2.87 kg [2.09, 3.65]). Nevertheless, this level of weight loss is of a similar order to the median of 2.8 kg (IQR 5.9, 0.7) reported in an evaluation of an NHS Weight Watchers referral scheme [25]. This suggests that FFIT for Women has promise as weight management intervention for women. It should also be noted that sex-based metabolic factors may partially account for the lower levels of weight loss observed in FFIT for Women, compared to the men’s FFIT programme [26], as might greater past attendance at other weight management programmes.

During FFIT, the median 12-week increase in total physical activity reported by men was 1484 MET-mins/week [12], compared with a smaller increase of 1140 MET-mins/week in FFIT for Women. While derived from a limited sample (30/123), the increase in physical activity observed in FFIT for Women is above the level advocated for health benefits and weight management in adults (i.e. 225–300 min of moderate intensity physical activity per week (or ~ 675–1799 MET-mins)) [27]. In addition, it is important to note that high levels of variability have been found when using the IPAQ to assess self-reported physical activity [28], and despite the apparent lower levels of physical activity benefit, qualitative data indicate that the inclusion of physical activity in FFIT for Women was seen by participants as a positive focus when compared to their experiences of commercial weight loss programmes. Both the in-programme physical activities and the pedometer-based walking programmes were (on the whole) met with enthusiasm. This may have been one of the ‘unique selling points’ of FFIT for Women, and one that could be more fully integrated into future weight management programmes aimed at women.

In comparison with differences seen at 12 weeks amongst the intervention group in the FFIT RCT, FFIT for Women participants reported somewhat higher reductions in fatty food (mean scores − 6.5 [− 4.4, − 8.56] v − 5.6 [− 4.86, − 6.39]) and sugary food scores (mean − 2.8 [− 1.86, − 3.77] v - 2.1 [− 1.84,–2.43]), but lower increases in fruit and vegetable scores (mean 1.2 [0.81, 1.66] v 1.6 [1.39, 1.81]), respectively. If these findings are borne out in larger samples, it may reflect greater prior familiarity with healthy eating messages amongst women, as reported in some attitude surveys [29], and thus less scope for improvement.

Aside from the benefits of losing weight and becoming more active, our qualitative data suggests that the psychological shifts may in part be due to the positive social environment that FFIT for Women provided; an environment in which many women said they felt less self-conscious about their bodies, and were able to give and receive support, and share ideas. For some, this was enhanced by the common bond of being fans of the host football club. Our findings are consistent with studies demonstrating the salience of supportive physical activity environments, whereby people with excess weight may experience an increased sense of safety, encouragement and social wellbeing [30, 31]. They are also consistent with evidence demonstrating that some people may avoid exercising in other settings due to perceived weight-related stigma and discrimination [32, 33].

Our study also suggests that women may benefit from more holistic approaches to weight management focusing on health behaviour change (i.e. physical activity and healthy eating) as opposed to rigid calorie restriction and long-term dieting. These findings resonate with alternative approaches to weight management that emphasise health and wellbeing as opposed to exclusively focusing on weight control [34].

Football clubs are increasingly being used to deliver weight management, health screening and other health improvement programmes. However, the extent to which programmes located within football clubs are able to produce meaningful outcomes for women is unclear [35]. While a small number of studies have documented that football clubs can reach older women [36, 37], these studies are reliant on self-reported and qualitative data and do not also report objectively-measured health outcomes. In this sense, the present study is unique and offers the first contribution to the literature on football setting-based interventions aimed specifically at women that provides some objectively-measured outcomes. While the setting was not the main appeal for all participants, it is clear from the qualitative data and the survey data, that a substantial sub-group had strong ties to the football club at which the programme was run.

This feasibility study has demonstrated that FFIT for Women shows a great deal of promise as a weight management programme, but it does have limitations. First, the before and after design, as opposed to a randomised comparison, was a product of the commissioning process and the only practical option for this feasibility study. This means that the standard of evidence produced by the study is lower than that produced for the FFIT programme (in the FFIT Pilot RCT). Related to this, the sample size for the study was also determined by the terms of the funders’ brief. Second, the percentage of women for whom post-programme weight data were available was just under 60%, although our analysis suggests that there were no statistically significant differences in baseline measures between those followed up and those lost to follow up. Thirdly, after cleaning and checking, self-report IPAQ data on physical activity was limited to 30 usable responses, which is less than half of those from whom follow-up measurements were collected. Finally, the FFIT for Women programme did not undergo the process of evidence-based gender-sensitisation that was undertaken during the development of FFIT. This may be a partial explanation of lower levels of weight loss at 12 weeks for FFIT for women than was found for men in the FFIT RCT.

Future research should look to improve on the promise that FFIT for Women has shown. Additional qualitative work should be done to explore how best to frame FFIT for Women for a target group which often includes those who have extensive experience of other weight loss programmes and healthy lifestyle messages. Work should also be done to consider how, and to what extent, to acknowledge that menstrual cycles may need to be considered as a barrier to some women in making behavioural changes. Further, future work should also explore the inclusion of feedback on alternative measures of body composition (other than weight or waist), described as having more salience for women specifically. A refined program would draw on this work, would improve attrition rates, and may facilitate progression to a definitive trial.

## Conclusion

As women’s presence in the field of football continues to grow, both as fans and as players, the promise of the club setting for health improvement programmes and initiatives needs to be explored more thoroughly. The dearth of evidence speaks to this need, and the present study makes an important contribution to this effort. Our evaluation of FFIT for Women has demonstrated, within the limits of pre- and post- test methodology, that positive outcomes are obtainable in this setting. Through a programme delivered in professional football club settings, women can lose weight, become more active, eat a better diet and feel better within themselves. Given that the extensive gender-sensitisation techniques that went into developing FFIT (for men) were not deployed in FFIT for Women, the outcomes reported here are promising but suggest that further sensitisation for women could lead to the development of a more effective weight-loss intervention.

In summary, FFIT for Women succeeded in attracting women at high risk of ill health, due to their weight. Attendance at programme sessions was good, but the football club setting was not seen as such a big attraction as for men taking part in FFIT, despite much of the cohort reporting regularly attending and viewing football matches. The programme supported women to make positive changes to their physical activity and eating habits, and to feel better in themselves. Mean weight loss was not as great as that achieved in men, but the programme still delivered a mean weight loss of 2.87 kg amongst those that were retained for post-programme measures. Additional tailoring of the content and style of delivery may help to improve weight loss outcomes, retention and acceptability.

## Additional files

Additional file 1: Study questionnaire. (PDF 1280 kb)

Additional file 2: Baseline differences between participants followed up at 12-weeks and participants lost to follow up. (DOCX 21 kb)

## Abbreviations

BMI: Body Mass Index
FFIT: Football Fans in Training
PANAS: Positive and Negative Affect Score
RCT: Randomised Controlled Trial
SPFLT: Scottish Professional Football League Trust
UEFA: Union of European Football Associations
