# Supplementary material for: Can professional football clubs deliver a weight management programme for women: a feasibility study
Source: BMC Public Health. 2018 Dec 3;18:1330. doi: 10.1186/s12889-018-6255-2 (PMC6276211; doi:10.1186/s12889-018-6255-2)
Supplement: Supplementary file 1 — Study questionnaire. (PDF 1280 kb) [file 12889_2018_6255_MOESM1_ESM.pdf]

FOOTBALL FANS IN TRAINING For Women

Women's fitness and healthy living programme

for

Aberdeen Football Club Supporters

SELF COMPLETE QUESTIONNAIRE

Section A: How you heard about FFIT and some questions about football

1

We are interested in finding out how people hear about the Football Fans in Training for Women Programme AND about the club that you support:

1a Can you please tell us how you found out about FFIT for Women.
Tick all boxes that apply:

- ☐ Saw an advert in a match programme
- ☐ Saw an advert on Facebook
- ☐ Heard about it on Twitter
- ☐ Read about it in a **club newsletter**
- ☐ Saw information about the programme **online/on a website**

  Please specify which website(s)
- ☐ Received an email about it

  Please specify who sent the email
- ☐ Heard about it from **someone else**

  Please say who
- ☐ **Other**

  Please say what

1b Can you tell us why you want to join the FFIT for Women Programme?

- [ ] To get **fitter**
- [ ] To lose **weight**
- [ ] To get **fitter for a specific reason**

  Please specify your reason

- [ ] To **improve my lifestyle**
- [ ] **Health reasons**

  Please specify your reason

- [ ] **Someone recommended** it to me

  Please specify who

- [ ] **Someone told me that I needed to go on it…**

  Please say who

- [ ] Because it was **at the club**
- [ ] Because it would be with **women like me**
- [ ] **Other**

  Please say what

Which of the above reasons was the most important?

Please write your answer below

Now, some questions about football!

2a Which of the following football clubs do you support, if any?
Please tick ONE box

Aberdeen ☐	Celtic ☐	Kilmarnock ☐	Motherwell ☐
Rangers ☐	Other (please specify opposite) ☐ →	____________________	

2b How far away is the home ground of the team you support from your home?
Please tick ONE box

Less than a mile ☐	2-3 Miles ☐	4-5 Miles ☐	6-10 Miles ☐
More than 10 Miles ☐			

2c When you go to a home game of the team you support, how do you usually travel there?
Please tick ONE box

Walk most / all of the way ☐	Get the Bus ☐	Go by Car ☐	Other ☐

During the football season:

3a How often do you go to the HOME games of the team you support?
Please tick ONE box

I go them all	I go to most of them	I go to some of them	I don't go to any of them
☐	☐	☐	☐

3b How often do you go to the AWAY games of the team you support?
Please tick ONE box

I go them all	I go to most of them	I go to some of them	I don't go to any of them
☐	☐	☐	☐

3c How often do you watch live or recorded football games on TV at home with friends or family?
Please tick ONE box

Every day	5-6 time a week	3-4 times a week	1-2 times a week
☐	☐	☐	☐
Occasionally	Never		
☐	☐		

3d How often do you go to the pub to watch a football game? Please tick ONE box

Every day	5-6 time a week	3-4 times a week	1-2 times a week
☐	☐	☐	☐
**Occasionally**	**Never**		
☐	☐		

4 A lot of people enjoy having a drink when watching football. How much would you drink?

	**BEER, LAGER or CIDER**<br>*Record number of pints*<br>330ml bottle = ½ pint<br>500ml can = 1 pint	**WINE**<br>*Record number of glasses*<br>1 bottle wine = 6 glasses	**SPIRITS**<br>*Record number of measures*<br>1 bottle spirits = 27 measures<br>¼ bottle = 7 measures	**OTHER**<br>(specify) ✎...
**before and after a <u>home match</u>?**<br>put 0 if none	✎...<br>PINTS	✎...<br>GLASSES	✎...<br>MEASURES	✎...
**if you go to the pub to watch a football game?**<br>put 0 if none	✎...<br>PINTS	✎...<br>GLASSES	✎...<br>MEASURES	✎...
**if you are watching a football game on TV at home or a friend's house?**<br>put 0 if none	✎...<br>PINTS	✎...<br>GLASSES	✎...<br>MEASURES	✎...

5 Have much have you done any of the following over the last 3 months?…

Please tick ONE box on EACH line	*Not at all*	*1-2 times a month*	*About weekly*	*Everyday or most days*
a tried to limit what you eat or drink to try to lose weight?	☐	☐	☐	☐
b done an exercise workout (including video/DVD workouts) at home?	☐	☐	☐	☐
c attended a commercial weight loss programme (e.g. Weight Watchers)	☐	☐	☐	☐
d attended a gym, leisure centre or local sport facility to swim or take part in other physical activity sessions?	☐	☐	☐	☐
e attended a weight-reduction clinic at your GP surgery or another NHS setting?	☐	☐	☐	☐

6 Is there anything else you have done over the last 3 months to try to lose weight ? ...
Please tick ONE box

Yes ☐ **If yes, please specify** ✐ ______________________

No ☐

Section B: Questions about your health and wellbeing, eating patterns, activity levels and about you.

We are interested in finding out about the kinds of physical activities that people do as part of their everyday lives. The questions will ask you about the time you spent being physically active in the **LAST 7 DAYS**. Please think about the activities you do at work, as part of your house and garden work, to get from place to place, and in your spare time for recreation, exercise or sport.

Please answer each question even if you do not consider yourself to be an active person.

Please think about the activities you do as part of everyday life and only those you did for at least 10 minutes at one time.

1 During the LAST 7 DAYS, on how many days did you do vigorous physical activities like heavy lifting, digging, aerobics or fast bicycling?

Think only of activities that you did for at least 10 minutes at one time and that took hard physical effort and made you breathe much harder than normal.
Write in days per week or tick if none.

A _________ **days per week (Go to B↓)**

or **none** ☐ **Go to ↓ Question 2**

B How much time in total did you usually spend on one of those days doing vigorous physical activities?
Write in hours & minutes EACH DAY or tick if don't know/not sure

_________ hours _________ minutes

or ☐ don't know/not sure

2 During the LAST 7 DAYS, on how many days did you do moderate physical activities like carrying light loads, bicycling at a regular pace, or doubles tennis? Do not include walking.

Think only of activities that you did for at least 10 minutes at one time and that took moderate physical effort and made you breathe somewhat harder than normal.
Write in days per week or tick if none

A _________ **days per week (Go to B ↓)**

or **none** ☐ **Go to Question 3 ➔**

B How much time in total did you usually spend on one of those days doing <u>moderate</u> physical activities?

Write in hours & minutes EACH DAY or tick if don't know/not sure

_________ hours _________ minutes

or ☐ don't know/not sure

3 During the LAST 7 DAYS, on how many days did you <u>walk</u> for at least 10 minutes at a time? This includes walking at work and at home, walking to travel from place to place, and any other walking that you did solely for recreation, sport, exercise or leisure.

Write in days per week or tick if none

A _________ days per week (Go to B↓)

or **none** ☐ **Go to Question 4 ↓**

B How much time in total did you usually spend walking on one of those days?

Write in hours & minutes ON ONE OF THOSE DAYS or tick if don't know/not sure

_________ hours _________ minutes

or ☐ don't know/not sure

The next question is about the time you spent sitting on weekdays while at work, at home, while doing course work and during leisure time. This includes time spent sitting at a desk, visiting friends, reading travelling on a bus or sitting or lying down to watch television.

4 During the LAST 7 DAYS, how much time in total did you usually spend <u>sitting</u> on a week day?

Write in hours & minutes ON ONE OF THOSE DAYS or tick if don't know/not sure

_________ hours _________ minutes

or ☐ don't know/not sure

5 **Comparing yourself with most people your age, would you rate your level of fitness as…** Please tick ONE box

very good	good	moderate	poor	very poor
☐	☐	☐	☐	☐

6 **Please tick one box on each line below to show whether you strongly agree, agree, disagree or strongly disagree with each statement.**

Please tick ONE box on EACH LINE

		**Strongly Agree**	**Agree**	**Disagree**	**Strongly Disagree**
1	On the whole, I am satisfied with myself.	☐	☐	☐	☐
2	At times, I think I am no good at all.	☐	☐	☐	☐
3	I feel that I have a number of good qualities.	☐	☐	☐	☐
4	I am able to do things as well as most other people.	☐	☐	☐	☐
5	I feel I do not have much to be proud of.	☐	☐	☐	☐
6	I certainly feel useless at times.	☐	☐	☐	☐
7	I feel that I'm a person of worth, at least on an equal plane with others.	☐	☐	☐	☐
8	I wish I could have more respect for myself.	☐	☐	☐	☐
9	All in all, I am inclined to feel that I am a failure.	☐	☐	☐	☐
10	I take a positive attitude toward myself.	☐	☐	☐	☐

THE NEXT FEW QUESTIONS ASK ABOUT YOUR HEALTH

7 In general, would you say your health is:
Please tick ONE box

Excellent	Very Good	Good	Fair	Poor
☐	☐	☐	☐	☐

8 Does this normally limit your activities in any way?
Please tick ONE box

a very great deal	quite a lot	to a moderate degree	only a little	not at all
☐	☐	☐	☐	☐

THE FOLLOWING TWO QUESTIONS ARE ABOUT ACTIVITIES YOU MIGHT DO DURING A TYPICAL DAY

Does YOUR HEALTH NOW LIMIT YOU in these activities?
If so, how much?

9A MODERATE ACTIVITIES, such as moving a table, pushing a vacuum cleaner, bowling, or playing golf:
Please tick ONE box

Yes, limited a lot ☐ Yes, limited a little ☐ No, not limited at all ☐

9B Climbing SEVERAL flights of stairs:

Please tick ONE box

Yes, limited a lot	Yes, limited a little	No, not limited at all
☐	☐	☐ 3

10 During the PAST 4 WEEKS have you had any of the following problems with your work or other regular activities AS A RESULT OF YOUR PHYSICAL HEALTH?

Please tick ONE box for each question

	Yes	No
ACCOMPLISHED LESS than you would like	☐	☐
Were limited in the KIND of work or other activities	☐	☐

11 During the PAST 4 WEEKS, have you had any of the following problems with your work or other regular daily activities AS A RESULT OF ANY EMOTIONAL PROBLEMS (such as feeling depressed or anxious)?

Please tick ONE box for each question	Yes	No
ACCOMPLISHED LESS than you would like	☐	☐
Didn't do work or other activities as CAREFULLY as usual	☐	☐

12 During the PAST 4 WEEKS, how much did PAIN interfere with your normal work (including both work outside the home and housework)?

Please tick ONE box

Not At All	A Little Bit	Moderately	Quite a Bit	Extremely
☐	☐	☐	☐	☐

13 The next three questions are about how you feel and how things have been during the PAST 4 WEEKS. For each question, please tick the one answer for each question that comes closest to the way you have been feeling.

How much of the time during the PAST 4 WEEKS…

Please tick ONE box on each line

	All of the time	Most of the time	A good bit of the time	Some of the time	A little of the time	None of the time
Have you felt calm and peaceful?	☐	☐	☐	☐	☐	☐
Did you have a lot of energy?	☐	☐	☐	☐	☐	☐
Have you felt downhearted and low?	☐	☐	☐	☐	☐	☐

14 During the PAST 4 WEEKS, how much of the time have your PHYSICAL HEALTH or EMOTIONAL PROBLEMS interfered with your social activities (like visiting friends, relatives, etc.)?

Please tick ONE box

All of the time	Most of the time	A good bit of the time	Some of the time	A little of the time	None of the time
☐	☐	☐	☐	☐	☐

15 Now, thinking about yourself and how you normally feel, to what extent do you generally feel….

Please one box on each line

	1 (never)	2	3	4	5 (always)
Upset	☐	☐	☐	☐	☐
Hostile	☐	☐	☐	☐	☐
Alert	☐	☐	☐	☐	☐
Ashamed	☐	☐	☐	☐	☐
Inspired	☐	☐	☐	☐	☐
Nervous	☐	☐	☐	☐	☐
Determined	☐	☐	☐	☐	☐
Attentive	☐	☐	☐	☐	☐
Afraid	☐	☐	☐	☐	☐
Active	☐	☐	☐	☐	☐

16 Please look at the list of NHS Services below.

Please tick NO or YES. If you tick 'yes' for any of the services, please give the number of times you have used the service in the LAST 3 MONTHS.

The example shows: 3 visits to the Dentist in last 3 months.

OVER THE LAST 3 MONTHS, have you used any of the following NHS Services?

Service	No	Yes		Number of visits
EXAMPLE: Dentist	No ☐	Yes ☑	→	3
Your GP or another GP	No ☐	Yes ☐	→	☐☐
Nurse	No ☐	Yes ☐	→	☐☐
Physiotherapist - outpatient	No ☐	Yes ☐	→	☐☐
Doctor or nurse in an emergency department *(casualty / A&E)*	No ☐	Yes ☐	→	☐☐

Hospital specialist – outpatient *(please specify)*

Specialist	No	Yes		Number of visits
✏...	No ☐	Yes ☐	→	☐☐
✏...	No ☐	Yes ☐	→	☐☐
✏...	No ☐	Yes ☐	→	☐☐

Hospital inpatient stay *(please specify)*

Number of visits

✏... No ☐ Yes ☐ → ☐☐

✏... No ☐ Yes ☐ → ☐☐

✏... No ☐ Yes ☐ → ☐☐

Other NHS Service *(please specify)*

Number of visits

✏... No ☐ Yes ☐ → ☐☐

✏... No ☐ Yes ☐ → ☐☐

✏... No ☐ Yes ☐ → ☐☐

17 In the LAST 3 MONTHS, please tell us if you have used any of these medications - either prescribed for you by a doctor or bought (by you or someone else on your behalf) without a prescription?

The example shows: Your Doctor had prescribed you eye drops ONCE and you also bought eye drops from the Chemist or other shops another FIVE times in the last 3 months.

In the LAST 3 MONTHS...	DOCTOR PRESCRIBED		BOUGHT WITHOUT A PRESCRIPTION (by you or someone else) from a Chemist or other shop	
		Number of times		Number of times
EXAMPLE: Eye drops	No ☐	Yes ☑ → 3	No ☐	Yes ☑ → 1
**Pain killers**	No ☐	Yes ☐ → ☐☐	No ☐	Yes ☐ → ☐☐
**Anti-inflammatory drugs** ***(eg: ibuprofen)***	No ☐	Yes ☐ → ☐☐	No ☐	Yes ☐ → ☐☐
**Gels / creams** ***(eg: ibuleve)***	No ☐	Yes ☐ → ☐☐	No ☐	Yes ☐ → ☐☐
**Inhalers for asthma**	No ☐	Yes ☐ → ☐☐	No ☐	Yes ☐ → ☐☐
**Sleeping pills**	No ☐	Yes ☐ → ☐☐	No ☐	Yes ☐ → ☐☐
**Anti-depressants**	No ☐	Yes ☐ → ☐☐	No ☐	Yes ☐ → ☐☐

18 Are you taking any other medications?

No ☐ Go to Q20 ↓ Yes ☐ Go to Q19 ↓

19 If 'Yes', please write the name(s) of the medications below and indicate the number of times that this has been prescribed or bought for you IN THE LAST 3 MONTHS.

In the LAST 3 MONTHS...	DOCTOR PRESCRIBED			BOUGHT WITHOUT A PRESCRIPTION (by you or someone else) from a Chemist or other shop		
			Number of times			Number of times
✏...	No ☐	Yes ☐ →	☐☐	No ☐	Yes ☐ →	☐☐
✏...	No ☐	Yes ☐ →	☐☐	No ☐	Yes ☐ →	☐☐
✏...	No ☐	Yes ☐ →	☐☐	No ☐	Yes ☐ →	☐☐
✏...	No ☐	Yes ☐ →	☐☐	No ☐	Yes ☐ →	☐☐
✏...	No ☐	Yes ☐ →	☐☐	No ☐	Yes ☐ →	☐☐
✏...	No ☐	Yes ☐ →	☐☐	No ☐	Yes ☐ →	☐☐

20 Below you will find a number of words or phrases which might describe someone's personality. We would like you to tell us how well each of the words describe <u>you</u>

Use the seven point scale to show us how well each word describes you.

Example: if the word was FRIENDLY

Write 6 in the box if it is usually true that you are friendly ➔ FRIENDLY ☐

REMEMBER TO FILL IN A BOX FOR EVERY WORD OR PHRASE

1	2	3	4	5	6	7
Never or almost never true	Usually not true	Sometimes but infrequently true	Occasionally true	Often true	Usually true	Always or almost always true

DEFEND MY OWN BELIEFS	☐	HAVE LEADERSHIP ABILITIES	☐
AFFECTIONATE	☐	EAGER TO SOOTHE HURT FEELINGS	☐
CONSCIENTIOUS	☐	SECRETIVE	☐
INDEPENDENT	☐	WILLING TO TAKE RISKS	☐
SYMPATHETIC	☐	WARM	☐
MOODY	☐	ADAPTABLE	☐
ASSERTIVE	☐	DOMINANT	☐
SENSITIVE TO NEEDS OF OTHERS	☐	TENDER	☐
RELIABLE	☐	CONCEITED	☐
STRONG PERSONALITY	☐	WILLING TO TAKE A STAND	☐
UNDERSTANDING	☐	LOVE CHILDREN	☐
JEALOUS	☐	TACTFUL	☐
FORCEFUL	☐	AGGRESSIVE	☐
COMPASSIONATE	☐	GENTLE	☐
TRUTHFUL	☐	CONVENTIONAL	☐

The next section looks at what you may have eaten and drunk over the **LAST 7 DAYS**. Please read each question carefully, ticking appropriate box for each option.

21 About how many times OVER THE LAST 7 DAYS did you eat breakfast?

No times	1-2 times	3-5 times	6 or more times
☐	☐	☐	☐

22 About how many times OVER THE LAST 7 DAYS did you eat/drink a serving of the following?

	No times	1-2 times	3-5 times	6 or more times
**Cheese** *(any except cottage)*	☐	☐	☐	☐
**Beef burgers or sausages**	☐	☐	☐	☐
**Beef, pork or lamb** *(if vegetarian: nuts)*	☐	☐	☐	☐
**Fried food** *(fried fish, cooked breakfast)*	☐	☐	☐	☐
**Chips**	☐	☐	☐	☐
**Bacon, processed meat**	☐	☐	☐	☐
**Pies, quiches, pastries**	☐	☐	☐	☐
**Crisps**	☐	☐	☐	☐
**Fast foods** *(takeaway or sit in)*	☐	☐	☐	☐

23 Thinking about THE LAST 7 DAYS: about how many times a day did you eat the following:

	Less than once a day	1-2 times a day	3-5 times a day	6 or more times a day
**Fruit and vegetables** *(not potatoes)*	☐	☐	☐	☐
**Chocolate, sweets**	☐	☐	☐	☐
**Biscuits**	☐	☐	☐	☐
**Sugary drinks** (fizzy drinks, diluting juice, fruit juice)	☐	☐	☐	☐

24 Thinking about THE LAST 7 DAYS: about how much milk did you use in a day, for drinking or in cereal, tea or coffee.

Less than a quarter pint	About a quarter pint	About half a pint	1 pint or more
☐	☐	☐	☐

25 What kind of milk do you usually use?

Full cream (blue top)	Semi skimmed (green top)	Skimmed (red top)
☐	☐	☐

Thinking about THE LAST 7 DAYS:
how much alcohol have you had to drink each day.

26 A Please circle which day is <u>TODAY</u>

Mon Tues Wed Thurs Fri Sat Sun

26 B Starting with yesterday and work back through the week, record the number of pints, glasses etc you had each day.

	RECORD IN **PINTS**	RECORD IN **GLASSES** 1 bottle wine = 6 glasses 1 bottle sherry = 12 glasses		RECORD IN **MEASURES** 1 bottle spirits = 27 measures ¼ bottle = 7 measures	
	**BEER LAGER CIDER**	**WINE**	**FORTIFIED WINE**	**SPIRITS**	**OTHER** (specify)
	PINTS	GLASSES	GLASSES	MEASURES	
MONDAY					
TUESDAY					
WEDNESDAY					
THURSDAY					
FRIDAY					
SATURDAY					
SUNDAY					

27A Have you ever smoked cigarettes?

☐ 2

☐ 3

☐ 1

If yes, how many do you usually smoke per day? ______

27B Do you ever smoke…?

Cigars ☐ ☐

Pipe ☐ ☐

This last section asks you for a few details about you and your current circumstances. This is so that we can see how women who take part in the FFIT for Women programme compare with all other women in Scotland.

28 **How old are you?**
✏...

		years old

29 **Are you?**

single, that is never married	☐ 1
married and living with husband	☐ 2
married but separated from husband	☐ 3
living with someone as a couple (but not married)	☐ 4
divorced	☐ 5
widowed	☐ 6

30 Are you living in a home which you...?

Own outright	☐
Are buying with the help of a mortgage or loan	☐
Pay part rent and part mortgage (shared ownership)	☐
Rent	☐
Live rent free (including rent free in relative's/friend's property)	☐
Other (please specify and tick)	☐

DATA ENTRY CODE

31 What is your highest educational qualification?

No educational qualifications	☐
Standard grades, O grades, O levels, GCE/GCSEs	☐
Highers, advanced highers, A levels	☐
Vocational qualification (e.g. SVQ/SCOTVEC)	☐
HNC/HND	☐
Degree (e.g. BA, BSc)	☐
Post-graduate qualification (e.g. MSc, PhD)	☐
Other (please specify and tick)	☐

DATA ENTRY CODE

32 Which ethnic background do you consider yourself to belong to?

Please tick ONE box

White

- British ☐
- Scottish ☐
- Irish ☐
- Any other white background ☐

Black or Black British

- Caribbean ☐
- African ☐
- Any other Black background ☐

Mixed

- White and Black Caribbean ☐
- White and Black African ☐
- White and Asian ☐
- Any other Mixed background ☐

Chinese or other ethnic group:

- Chinese ☐
- Any other (please tick and specify below) ☐

✏...
......

Asian or Asian British

- Indian ☐
- Pakistani ☐
- Bangladeshi ☐
- Any other Asian background ☐

33 Which of these descriptions best describes what you were doing last week?

Please tick ONE box

- In paid employment or self-employed (or temporarily away) ☐
- Doing unpaid work for a business that you own, or that a relative owns ☐
- Waiting to take up paid work already obtained ☐
- On a Government scheme for employment training ☐
- Looking for paid work or a Government training scheme ☐
- Intending to look for work but prevented by temporary sickness or injury ☐
- Permanently unable to work because of long-term sickness or disability ☐
- Going to college full-time (including on holiday) ☐
- Retired from paid work ☐
- Looking after home or family ☐
- Doing something else (please specify and tick) ☐

DATA ENTRY CODE

Thank you for completing these questions
A researcher will now help you complete the few next questions and measurements

34 Food Portion Station

Ask the man to look at the pictures and decide which portion most resembles what he currently eats. Record the number of the portion (1-8) against each type of food listed.

**Cheese** ✏		**Meat** ✏	
**Pasta** ✏		**Chips** ✏	

35 **ASK:** do you have a long-standing illness, disability or infirmity?

By long-standing we mean anything that has troubled you over a period of time or that is likely to affect you over a period of time?

Yes ☐ No ☐

36 What is the matter with you?

(write BRIEF description of all LONG-STANDING conditions mentioned)

Does this normally limit your activities in any way?

Please tick **one** box for **each** listed condition

	A very great deal	Quite a lot	To a moderate degree	Only a little	Not at all
1 ✏...	☐	☐	☐	☐	☐
2 ✏...	☐	☐	☐	☐	☐
3 ✏...	☐	☐	☐	☐	☐
4 ✏...	☐	☐	☐	☐	☐

37 INJURIES

37a Do you have any current or past injuries that affect your ability to undertake usual activities or exercise?

Please tick **one** box

Yes ☐ 1 → **Q37b**

No ☐ 2 → **check**

37b How many injuries affect your activities?

Please tick **one** box

If more than 3, record 3 most limiting injuries

1 ☐ provide details @ injury 1

2 ☐ provide details @ injury 1 & 2

3+ ☐ provide details @ injury 1, 2 & 3

	**Injury 1 ↓**			**Injury 2 ↓**			**Injury 3 ↓**		
**Brief description of injury**									
**What type of injury** Please circle Y/N on **each line**	Break or fracture	Y_1	N_2	Break or fracture	Y_1	N_2	Break or fracture	Y_1	N_2
	Ligament / Cartilage / Muscle damage	Y_1	N_2	Ligament / Cartilage / Muscle damage	Y_1	N_2	Ligament / Cartilage / Muscle damage	Y_1	N_2
	Dislocation of joint	Y_1	N_2	Dislocation of joint	Y_1	N_2	Dislocation of joint	Y_1	N_2
	Severe bruising / sprain	Y_1	N_2	Severe bruising / sprain	Y_1	N_2	Severe bruising / sprain	Y_1	N_2
	Other (specify)	Y_1	N_2	Other (specify)	Y_1	N_2	Other (specify)	Y_1	N_2
**Date of injury**	M M Y Y			M M Y Y			M M Y Y		
**Location of injury** Please circle Y/N on **each line**	Lower Limbs Hip, Knee, Leg, Ankle, Foot, Toes	Y_1	N_2	Lower Limbs Hip, Knee, Leg, Ankle, Foot, Toes	Y_1	N_2	Lower Limbs Hip, Knee, Leg, Ankle, Foot, Toes	Y_1	N_2
	Upper Limbs Shoulder, Arm, Elbow, Hand, Fingers	Y_1	N_2	Upper Limbs Shoulder, Arm, Elbow, Hand, Fingers	Y_1	N_2	Upper Limbs Shoulder, Arm, Elbow, Hand, Fingers	Y_1	N_2
	Torso/ Upper body Back, Neck, Ribs	Y_1	N_2	Torso/ Upper body Back, Neck, Ribs	Y_1	N_2	Torso/ Upper body Back, Neck, Ribs	Y_1	N_2
	Other (specify)	Y_1	N_2	Other (specify)	Y_1	N_2	Other (specify)	Y_1	N_2
**Did this injury require…** Please circle Y/N on **each line**	Limitation of usual activities	Y_1	N_2	Limitation of usual activities	Y_1	N_2	Limitation of usual activities	Y_1	N_2
	Hospital treatment	Y_1	N_2	Hospital treatment	Y_1	N_2	Hospital treatment	Y_1	N_2
	Physiotherapy	Y_1	N_2	Physiotherapy	Y_1	N_2	Physiotherapy	Y_1	N_2
	Other treatment	Y_1	N_2	Other treatment	Y_1	N_2	Other treatment	Y_1	N_2
**To what extent does this injury still limit your day to day activities** Please circle Y/N on **each line**	Difficulty walking	Y_1	N_2	Difficulty walking	Y_1	N_2	Difficulty walking	Y_1	N_2
	Difficulty climbing stairs	Y_1	N_2	Difficulty climbing stairs	Y_1	N_2	Difficulty climbing stairs	Y_1	N_2
	Difficulty doing physical activity	Y_1	N_2	Difficulty doing physical activity	Y_1	N_2	Difficulty doing physical activity	Y_1	N_2

38 JOINT PAIN

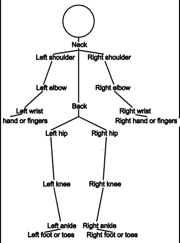

		Pain frequency<br>"How often do you get pain in your…?"					Pain severity<br>"Is the pain…?"					Limit to activities<br>"Does this limit your day to day activities…?"					
		All or most of the time	Only from time to time	Never	Don't Know		Severe	Moderate	Slight	Don't know		A very great deal	Quite a lot	To a moderate degree	Only a little	Or not at all	Don't know
Neck		1	2	3	9	→ complete severity & limits if freq. answer is 1, 2 or 9	1	2	3	9		1	2	3	4	5	9
Back		1	2	3	9		1	2	3	9		1	2	3	4	5	9
Shoulder	L	1	2	3	9	→ complete severity & limits if freq. answer is 1, 2 or 9	1	2	3	9		1	2	3	4	5	9
	R	1	2	3	9		1	2	3	9		1	2	3	4	5	9
Elbow	L	1	2	3	9	→ complete severity & limits if freq. answer is 1, 2 or 9	1	2	3	9		1	2	3	4	5	9
	R	1	2	3	9		1	2	3	9		1	2	3	4	5	9
Wrist	L	1	2	3	9	→ severity & limits if freq. answer is 1, 2 or 9	1	2	3	9		1	2	3	4	5	9
	R	1	2	3	9		1	2	3	9		1	2	3	4	5	9
Hand/ Finger	L	1	2	3	9	→ complete severity & limits if freq. answer is 1, 2 or 9	1	2	3	9		1	2	3	4	5	9
	R	1	2	3	9		1	2	3	9		1	2	3	4	5	9
Hip	L	1	2	3	9	→ complete severity & limits if freq. answer is 1, 2 or 9	1	2	3	9		1	2	3	4	5	9
	R	1	2	3	9		1	2	3	9		1	2	3	4	5	9
Knee	L	1	2	3	9	→ complete severity & limits if freq. answer is 1, 2 or 9	1	2	3	9		1	2	3	4	5	9
	R	1	2	3	9		1	2	3	9		1	2	3	4	5	9
Ankle	L	1	2	3	9	→ complete severity & limits if freq. answer is 1, 2 or 9	1	2	3	9		1	2	3	4	5	9
	R	1	2	3	9		1	2	3	9		1	2	3	4	5	9
Foot / Toes	L	1	2	3	9	→ complete severity & limits if freq. answer is 1, 2 or 9	1	2	3	9		1	2	3	4	5	9
	R	1	2	3	9		1	2	3	9		1	2	3	4	5	9

THANK MAN FOR COMPLETING THIS QUESTIONNAIRE

QUESTIONNAIRE CHECK

	IDNO			INITIALS		
**Questionnaire** (Checker)						
**S A H R** (Completer)						

University of Glasgow | Institute of Health & Wellbeing
MRC Medical Research Council
Aberdeen FC Football Club 1903
SPFL TRUST Scottish Football Supporting Communities

MEASUREMENTS

FOOTBALL FANS IN TRAINING

DATE

		/			/		

BLOOD PRESSURE

TEMPERATURE

__ __ . __ °C

SITTING 5 MINS?	YES	NO
BP LETTER GIVEN?	YES	NO

SURVEY ASST. IDNO/ INITIALS

*If SYSTOLIC over **150 or** DIASTOLIC **over 90,** issue BP letter*

SYSTOLIC		DIASTOLIC	PULSE	ARM	OMRON ID	CUFF ID
	/					

If any problems with first reading – record again and state issue with first reading below.

	/					

IF MEASUREMENT NOT TAKEN OR ANY ISSUES - PLEASE RECORD HERE

WAIST

WAIST

SURVEY ASST. IDNO/ INITIALS

1 __ __ __ . __ CMS

2 __ __ __ . __ CMS

If difference between measures 1 and 2 is ≥ 0.5cm, record 3rd measure

3 __ __ __ . __ CMS

IF MEASUREMENT NOT TAKEN OR ANY ISSUES - PLEASE RECORD HERE

HEIGHT

STADIOMETER IDNO | SURVEY ASST. IDNO/ INITIALS

			.		CMS

IF MEASUREMENT NOT TAKEN OR ANY ISSUES (esp. balance, posture, wearing turban etc) - PLEASE RECORD HERE

WEIGHT

SCALES IDNO | SURVEY ASST. IDNO/ INITIALS

			.		KGS

IF MEASUREMENT NOT TAKEN OR ANY ISSUES - PLEASE RECORD HERE

DOCUMENT ENDS
