# Supplementary material for: Can professional football clubs deliver a weight management programme for women: a feasibility study
Source: BMC Public Health. 2018 Dec 3;18:1330. doi: 10.1186/s12889-018-6255-2 (PMC6276211; doi:10.1186/s12889-018-6255-2)
Supplement: Supplementary file 2 — Baseline differences between participants followed up at 12-weeks and participants lost to follow up. (DOCX 21 kb) [file 12889_2018_6255_MOESM2_ESM.docx]

**Supplementary File 2: baseline differences between participants followed up at 12-weeks (n=72) and participants lost to follow up (n=51)**

|  | **All Participants**  **Mean±SD (N)** | **Followed Up**  **Mean±SD (N)** | **Lost to Follow Up**  **Mean±SD (N)** | **Independent Samples t-test *p*** |
| --- | --- | --- | --- | --- |
| **Physical measures** |  |  |  |  |
| Age (years) | 45.8±7.4 (123) | 46.5±7.1 (72) | 44.8±7.7 (51) | p=0.221 |
| Weight (kg) | 95.3±17.7 (123) | 93.2±16.9 (72) | 98.2±18.7 (51) | p=0.124 |
| BMI (kg/m^2^) | 36.6±6.9 (123) | 35.6±6.3 (72) | 37.9±7.6 (51) | p=0.066 |
| Waist (cm) | 105.1±12.4 (122) | 103.7±12.2 (72) | 107.0±12.5 (50) | p=0.147 |
| BP Systolic (mmHg) | 126.4±17.1 (116) | 128.3±16.8 (69) | 123.8±17.6 (47) | p=0.165 |
| BP Diastolic (mmHg) | 83.6±10.8 (116) | 84.2±11.3 (69) | 83.0±10.2 (47) | p=0.560 |
| **Employment**  **status** | **% (N)** | **% (N)** | **% (N)** | **X^2^  *p*** |
| In paid employment or self-employed | 84.6 (104) | 83.3 (60) | 86.3 (44) | p=0.754 |
| Permanently unable to work | 4.1 (5) | 5.6 (4) | 2 (1) |  |
| Retired from paid work | 2.4 (3) | 2.8 (2) | 2 (1) |  |
| Looking after home or family | 7.3 (9) | 6.9 (5) | 5.9 (3) |  |
| Other | 1.6 (2) | 1.4 (1) | 3.9 (2) |  |
| **Educational attainment** | **% (N)** | **% (N)** | **% (N)** | **X^2^  *p*** |
| No educational qualifications | 3.3 (2) | 5.6 (4) | 2.0 (1) | p=0.884 |
| Standard grades or equivalent | 19.5 (24) | 20.8 (15) | 18.0 (9) |  |
| Highers or equivalent | 10.6 (13) | 12.5 (9) | 12.0 (6) |  |
| Vocational qualification | 10.6 (13) | 6.9 (5) | 10.0 (5) |  |
| HNC/HND | 22 (27) | 19.4 (14) | 26.0 (13) |  |
| First degree | 20.3 (25) | 19.4 (14) | 20.0 (10) |  |
| Post-graduate qualification | 8.9 (11) | 11.1 (8) | 6.0 (3) |  |
| Other | 3.3 (4) | 4.2 (3) | 6.0 (3) |  |
| Missing | 1.6 (2) |  |  |  |
| **Marital Status** | **% (N)** | **% (N)** | **% (N)** | **X^2^  *p*** |
| Single | 16.3 (20) | 16.7 (12) | 16.0 (8) | p=0.326 |
| Married | 54.5 (67) | 59.7 (43) | 48.0 (24) |  |
| Separated | 1.6 (2) | 0.0 (0) | 4.0 (2) |  |
| Living with someone | 17.9 (22) | 16.7 (12) | 20.0 (10) |  |
| Divorced | 8.9 (11) | 6.9 (5) | 12.0 (6) |  |
| Missing | 0.8 (1) |  |  |  |
| **Housing Status** | **% (N)** |  |  | **X^2^  *p*** |
| Own outright | 17.9 (22) | 19.4 (14) | 16.0 (8) | p=0.651 |
| Mortgage or loan | 57.7 (71) | 58.3 (42) | 58.0 (29) |  |
| Rent | 22.0 (27) | 20.8 (15) | 24.0 (12) |  |
| Live rent free | 0.8 (1) | 1.4 (1) | 0.0 (0) |  |
| Other | 0.8 (1) | 0.0 (0) | 1.4 (1) |  |
| Missing | 0.8 (1) |  |  |  |
| **Ethnicity** | **% (N)** | **% (N)** | **% (N)** | **X^2^ *p*** |
| White British | 22.8 (28) | 23.6 (17) | 21.6 (11) | p=0.790 |
| White Scottish | 77.2 (95) | 76.4 (55) | 78.4 (40) |  |
